# Investigating the Adoption of Precautionary Behaviors Among Young Rural Adults in South Iran During COVID-19

**DOI:** 10.3389/fpubh.2022.787929

**Published:** 2022-02-02

**Authors:** Maryam Tajeri Moghadam, Tahereh Zobeidi, Stefan Sieber, Katharina Löhr

**Affiliations:** ^1^Department of Extension and Rural Development, Faculty of Agriculture, University of Tabriz, Tabriz, Iran; ^2^Department of Agricultural Extension, Communication and Rural Development, Faculty of Agriculture, University of Zanjan, Zanjan, Iran; ^3^Cooperation and Transformative Governance Research, International Institute for Applied Systems Analysis (IIASA), Laxenburg, Austria; ^4^Leibniz Center for Agricultural Landscape Research (ZALF), Müncheberg, Germany; ^5^Institut für Agrar- und Gartenbauwissenschaften, Humboldt-Universität zu Berlin, Berlin, Germany; ^6^Leibniz Centre for Agricultural Landscape Research (ZALF) e.V., Müncheberg, Germany

**Keywords:** COVID-19, preventive behavior, rural youth, health belief model, adoption

## Abstract

COVID-19 is an unprecedented challenge for public health worldwide. Reducing the incidence of the disease requires protective measures to prevent virus transmission. Understanding those factors influencing preventive behavior is the first step in preventing the spread of the disease. This study investigates factors affecting youth intention and preventive behaviors in the face of COVID-19 through the health belief model by using a cross-sectional survey collected through an online questionnaire. The sample comprises 304 rural youth in South Iran who were selected through a random sampling technique. The results reveal that perceived severity, perceived benefits, public health beliefs, perceived self-efficacy, and the cue to act positively and significantly affect preventive behaviors. The model explains 59% of variance changes in rural youth preventive behaviors during COVID-19. Cue to action is the strongest and self-efficacy was the weakest determinant of youth's preventive behavior. This study confirms that the HBM framework has appropriate predictive power and is an effective tool for investigating preventive behaviors during COVID-19. These results provide important policy implications for the development of policies that aim to avoid the further spread of COVID-19 between young citizens.

## Introduction

On January 30, 2020, the World Health Organization (WHO) issued a statement declaring the outbreak of the new coronavirus to be a public health emergency and a threat to the entire world (1). Due to its contagious nature and rapid spread around the world, on March 11, 2020, the World Health Organization confirmed it as a pandemic (2). The disease severely disrupted the daily activities of more than half of the world's population. The movement of people and many goods from different regions stopped completely (3). The coronavirus not only severely affected people's health, it also affected the world economy (4). The increasing outbreak of COVID-19 cases and deaths has also increased the fears of household vulnerability to food insecurity worldwide (5).

To counteract the rapid spread of the disease, key action areas were promoted by many governments, seeking to minimize virus transmission by promoting preventive behaviors that would flatten the peak of the disease and reduce its impact on health care, allowing doctors to treat severe cases while also reducing overall mortality (6). Existing research shows that the rapid spread of COVID-19 is strongly associated with the displacement of people with no-to-mild symptoms; i.e., those who are unaware they are infected (7). Therefore, non-medical practices, such as promoting protective behaviors like staying at home (home quarantine and reducing human interactions), increasing social distance, avoiding travels, and following personal hygiene tips (washing hands regularly and using masks when going to public places and crowded places) (8, 9), are used to control the spread of the disease (10). Promoting these preventive behaviors is essential for slowing the spread of COVID-19 (11, 12).

Despite the availability of COVID-19 prevention recommendations, people's adoption of behaviors that prevent the spread of COVID-19 differs between groups, generally leaving room for improvement. Monitoring people's perceptions and behavioral responses to COVID-19 is necessary to improve health risk communications and achieve successful changes in people's behaviors. However, globally, there is little information about people's perceptions and preventive behaviors regarding COVID-19.

Protective measures to prevent the transmission of the disease largely depend on rapid changes in the behavior of communities, thus relying on the ability of individuals to understand the risks associated with the virus and adapt their behaviors accordingly (13). For example, Wise et al. (13) show that understanding higher personal risk predicts preventive behaviors like hand washing and social distance. Akter (6) show that people who perceive a threat from COVID-19 collect medical and cleaning equipment, while those who do not take the disease seriously are careless, for example, moving around freely, traveling, gathering together, and not engaging in any preventive behaviors.

Reports indicate that rural communities perform fewer preventive health behaviors than urban communities. Villagers are not just more likely to have a negative attitude toward the effectiveness of preventive behaviors but also have a lower level of information about COVID-19 and its prevention measures (14). Studies also show that rural communities face health inequalities due to numerous barriers, including a lack of health care infrastructure (comprising transportation, health insurance, providers, and facilities), geographical distance, and lower socioeconomic status (15–17). The lack of these resources, services, and support put these populations at a higher risk and vulnerability (17). Public health researchers are concerned that rural communities may experience a worse situation in relation to the COVID-19 pandemic, for example, higher mortality rates, than their urban and suburban counterparts due to rural/urban health inequalities (18, 19).

In addition, reports indicate that rural communities, especially rural youth, are not taking the risk of the disease seriously and are continuing their daily activities. Further, people are usually reluctant to employ preventive behaviors due to physical discomfort, cost, and inconvenience (20). The results of an Italian study by Ceccato et al. (21) show that younger people are less confident in the information received about COVID-19 and are less interested in restrictive measures. They think that most deaths are related to the elderly or people with underlying diseases.

According to FAO reports (20), rural youth suffer disproportionately from the ubiquity of COVID-19 and its consequences because they are among the most vulnerable groups during the twenty-first century. Facing high unemployment rates, not only do rural youth have very little financial strength and income, but they also lack health insurance coverage and other social supports (20). Young villagers are vital stakeholders because they are in direct contact with food resources in supply chains. Their travel to urban areas and remaining there is in opposition to health advice and can increase the spread of the disease. For this reason, the protection of this group and the encouragement of healthy behaviors within it are of major importance. The study of health protection measures in Iran is important because it was the first low- or middle-income country to suffer a major outbreak including rural areas and learning from Iran's experience will help all low- and middle-income countries (22). Certainly in developing countries, including Iran, the shock of COVID-19 will be much more severe because most farmers in rural areas in these countries are not just prone to poverty and extreme vulnerability but they also have limited access to formal mechanisms to deal with these shocks (23).

Following these findings, this study investigates why the adoption of preventive behavior by youth differs from other age groups? This research seeks to answer this question among rural youth in Bushehr province of southern Iran. Iran and its southern region are chosen because of its severe experience with COVID-19 (5, 17, 24, 25). According to statistics, as of August 10, 2021, COVID-19 has resulted in 94,603 deaths in Iran, with numbers continuing to increase (26). The existing literature shows that various factors affect the preventive behavior of people with respect to infectious diseases. Further, the behaviors of people in potentially high-risk groups can play an important role in preventing and controlling infectious diseases (27). Thus, it is important to examine why some people, especially rural youth, participate in the prevention, screening, and control of health behaviors regarding COVID-19, while others do not. However, little information exists on the reasons for the participation or non-participation of rural youth in Iran with respect to preventive behaviors for COVID-19 and the evaluation of the factors affecting this behavior.

Given the significance of psychological and behavioral factors in the management of severe global pandemics, like COVID-19, it is essential to assess behavioral and psychological responses to the situation and to determine the relationship between perceived risk and participation in engaging in protective behaviors to facilitate disease minimization strategies (13). Studies use various theories and models to investigate the factors influencing preventive behaviors for various diseases, including COVID-19. For example, the health belief model to predict the use of personal protective equipment in SARS outbreaks (28), unified theory of acceptance and use of technology to predict the willingness to wear a mask (29), the theory of protection motivation against COVID-19 (22, 30), as well as knowledge, attitudes, and practices theory to investigate preventive behaviors against COVID-19 (27). In this regard, Mukhtar (31) recommends using the Health Beliefs Model (HBM) for recognizing preventive behaviors during the COVID-19. Since, HBM is one of the most functional theories related to behavioral change and preventive health behaviors (32), this theory is used in this study.

In addition, recent studies on the use of psychological structures and models in predicting COVID-19 prevention behaviors have focused on different groups of people. For example, study Irigoyen-Camacho et al. (33) in Mexocity on the behavior of staying at home among a group of adults, Roberts and David (34) on the general acceptance of preventive behaviors by undergraduate students in the United States, Šurina et al. (35) on compliance with health recommendations and social distance among the Latvian general population, and the study Lin et al. (36) on the acceptance of preventive behaviors by the people of Iran. However, in most of these studies, the rural youth group is neglected. Therefore, this study is innovative in two ways: first, an HBM theory has been used and second, it has considered rural youth. Therefore, in this study, the HBM framework, one of the most popular health research frameworks, is used to understand why rural youth take or do not take action for personal or community health in the face of COVID-19.

### Health Beliefs Model

**Figure 1:**
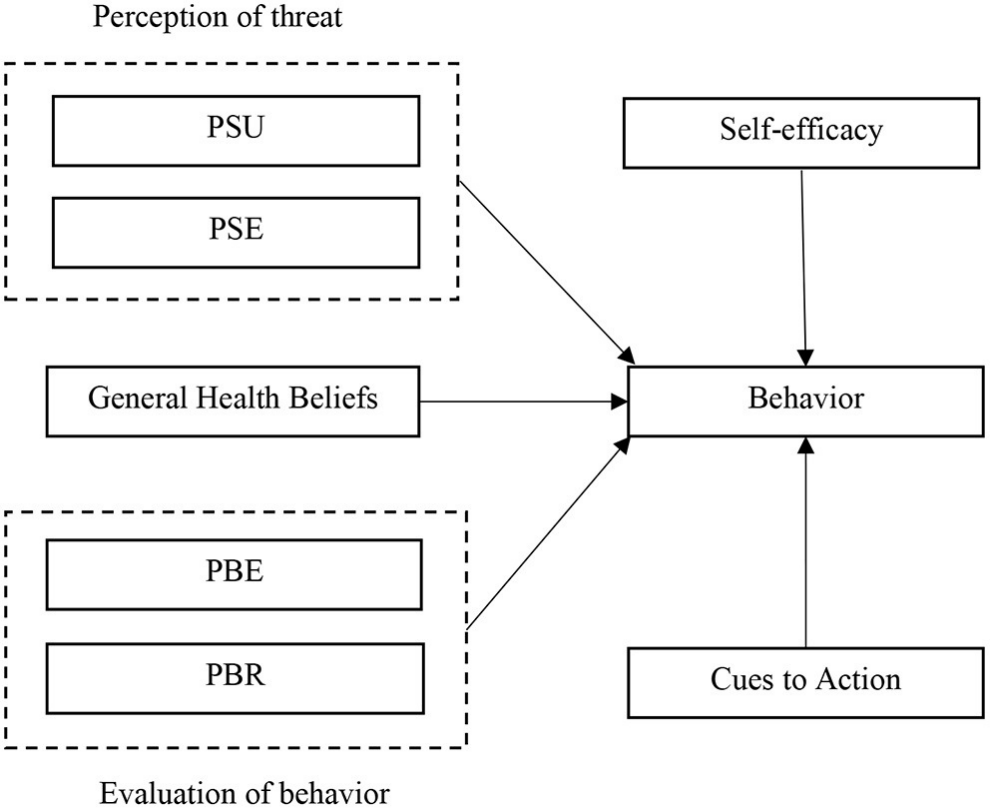
Health belief model.

HBM was introduced in the early 1950s by social psychologists in the United States (37). HBM assumes that safe actions originate from two main mechanisms: One's attitude toward danger (threat perception) and individual assessment of preventive behavior (behavioral evaluation). Perception of threat includes two sub-components or social psychological beliefs: perceived susceptibility (PSU) and perceived severity (PSE). The behavioral assessment comprises two sub-components of perceived benefits (PBE) and perceived barriers (PBR) (38, 39). PSU means people's beliefs regarding the probability of getting an illness (32). PSE means the belief that a severe health problem can lead to death or other serious consequences (40–42). The PBE refers to a person's assessment of the worth or effectiveness of performing health-oriented behaviors to reduce the risk of disease (37). Perceived barriers refer to a person's estimation of challenges to behavior change (43). HBM assumes that people engage in preventative behaviors if they (i) believe that they are more likely to get a disease (perceived susceptibility); (ii) understand the depth of the risk and its severity (perceived severity); (iii) perceive the benefits by adopting preventive health behaviors; and (iv) recognize that there is an imperfect obstacle to the implementation of preventive behaviors (44, 45) (see Figure 1).

In addition to these variables, HBM includes other variables that predict behavior, including self-efficacy, cue to action, and general health beliefs (46). Self-efficacy originates from Bandura's known theory (Social Cognitive Theory), which refers to the confidence and belief that the person can perform a specific behavior (47). HBM also states that a trigger is necessary to stimulate the conduct of health behaviors. Cues to action remind someone to engage in a particular behavior (48). Cue to action may be internal (physical symptoms of pain, fever, cough) or external (friends, health care providers, media, and social media) (49). In HBM, general health beliefs refer to the person's disposition or routine regarding health seeking behavior in general. It is not related to the expected concerns of healthcare behavior; instead, it is a person's general response predisposition (50).

Therefore, the present study examines seven following hypotheses.

H1) Perceived Susceptibility positively affects people's preventive behavior.
H2) Perceived severity positively affects people's preventive behavior.
H3) Perceived benefits positively affect people's preventive behavior.
H4) Perceived barriers negatively affect people's preventive behavior.
H5) Perceived self-efficacy positively affects people's preventive behavior.
H6) Cue to action positively affects people's preventive behavior.
H7) General health beliefs directly affect people's preventive behavior.

## Methodology

### Participants

The present study is applied research in terms of purpose, non-experimental in terms of control variables, and a field survey in terms of data collection. This study is quantitative research carried out through a sectional online survey. Data were collected in August 2020 through an online platform; all data were collected in the Persian language. The population of concentration (statistical population) of this study comprises rural youth (between 18 and 30 years) of Dashtestan county in Bushehr province in southern Iran (*N* = 1,500). Based on (51) table and using random sampling, 304 rural youths were selected as the study sample. The mean age of respondents was 24.79 years. In terms of gender, 125 people (41%) were male, and 180 were female (59%). The average household size was 4.68, with a standard deviation of 1.67.

### Survey

The data collection tool in this study was an online questionnaire. It consisted of two sections, including socio-economic characteristics and the HBM variables. HBM variables measured in the form of a spectrum of a 5-point Likert scale: “very low (1)“ to ”very high (5)" that were extracted from previous studies. Faculty members confirmed the facial and content validity and psychometric properties of the questionnaire. The internal validity of each variable examined using Cronbach's alpha. As shown in Table 1, all scales had good to excellent validity, generally between 0.78 and 0.93 (Table 1). SPSSversion24 and AMOSversion20 software used to analyze the data.

**Table 1:** Survey items.

**Constructs**	**References**
**Perceived susceptibility (PSU)** (α = 0.88) (Mean = 3.40) (SD = 1.05)	(8)
How likely is it that you will get corona if you go out shopping?	
How likely is it that if you go out to work or study, you will get corona?	
How likely is it that if you go out to visit family or friends, you will get corona?	
How likely is it that if you leave home for any other reason, you will get corona?	
**Perceived severity (PSE)** (α = 0.78) (Mean = 4.18) (SD = 0.71)	(8)
How dangerous do you think it is for you if you get corona?	
How much do you think it will cost you if you get corona?	
How much do you think will affect your life if you get corona?	
How much do you think will affect your family if you get corona?	
How much do you think will affect your work or education if you get corona?	
**General health beliefs** (α = 0.93) (Mean = 3.91) (SD = 0.90)	(40, 42)
I am inherently a healthy person and I always follow health issues.	
I am constantly mindful of prevention behavior against disease.	
I read information about safe behavior.	
**Perceived benefits (PBE)** (α = 0.78) (Mean = 4.14) (SD = 0.67)	(8, 22, 25)
The use of protective methods and devices prevents the spread of corona.	
The use of protection methods and devices prevents an epidemic in the village.	
The use of protection methods and equipment reduces the cost of the country.	
The use of protection methods and equipment reduces pressure on medical staff and hospitals.	
The use of protection methods and devices will save the lives of many of our compatriots.	
The use of protective methods and devices prevents the high cost of treatment.	
**Perceived barriers (PBR)** (α = 0.79) (Mean = 3.14) (SD = 0.75)	(8)
The use of these methods and protective devices is cumbersome.	
Using methods and protection devices makes work and activity difficult.	
I am not used to using these methods and protective devices.	
**Self-efficacy** (α = 0.76) (Mean = 3.95) (SD = 0.93)	(8, 52)
The use of protection methods and devices depends only on my own free will.	
Even if I want to, I cannot easily use these protective devices and methods.	
**Cues to action** (α = 0.89) (Mean = 4.17) (SD = 0.79)	(40, 42)
I've seen and heard a lot on TV and radio about the corona, and how to fight it.	
I've seen and heard a lot on social media, such as WhatsApp and Telegram, about the corona, and how fight it.	
I've heard a lot from friends and relatives about corona and how fight it.	
I have seen and heard a lot from officials and health experts about corona and ways to fight it.	
**Behavior** (α = 0.86) (Mean =) (SD =)	(6, 8, 31)
I stay home as much as possible and I do not go out.	
If I go out, I wear a mask.	
If I go out, I wear gloves.	
I do not touch people.	
I regularly use alcohol to disinfect my hands.	
I wash my hands regularly with soap and water.	
I disinfect and wash household items after purchase.	
I do not go to crowded and high risks places as much as possible.	

## Results

### Description and Relationship Between Variables

The Pearson correlation test is applied to examine the correlation among HBM constructs (Table 2). The results show that there is a positive and significant correlation between preventive behavior and perceived severity, PSU, PBE, self-efficacy, general health beliefs, and cue to action. The PBR is not meaningfully correlated to preventive behavior.

**Table 2:** Correlation matrix.

**Variables**	**Perceived**	**Perceived**	**Perceived**	**Perceived**	**Self-**	**general**	**Cue to**	**Behavior**
	**severity**	**susceptibility**	**benefit**	**barrier**	**efficacy**	**health beliefs**	**action**	
Perceived severity	1							
Perceived susceptibility	0.52^**^	1						
Perceived benefit	0.33^**^	0.29^**^	1					
Perceived barrier	−0.17^**^	−0.5	0.08	1				
Self-efficacy	0.14^*^	0.16^**^	0.37^**^	0.09	1			
General health beliefs	0.28^**^	0.19^**^	0.24^**^	−0.06	0.20^**^	1		
Cue to action	0.36^**^	0.20^**^	0.37^**^	−0.004^**^	0.23^**^	0.53^**^	1	
Behavior	0.37^**^	0.29^**^	0.44^**^	0.05	0.33^**^	0.52^**^	0.57^**^	1
CR	0.799	0.890	0.813	0.802	0.783	0.983	0.899	0.867
AVE	0.449	0.669	0.428	0.516	0.550	0.836	0.689	0.452

* *P < 0.05.*

** *P < 0.01*.

### Main Analysis

Structural equation modeling is used to investigate the explanatory power of HBM in predicting preventive behaviors. The two stages of structural equation modeling include Confirmatory Factor Analysis (CFA) to evaluate the suitability of the measurement model and structural equation modeling (53).

### Assessment of Measurement Model

To estimate the measurement models of the components affecting preventive behavior, the data collected using AMOS 20 was analyzed using CFA. The results show the measurement model has an acceptable fit. (RMSEA = 0.038; CMIN = 796.705, DF = 550, *p* = 0.000; CMIN/DF = 1.449; CFI = 0.957; GFI = 0.878; AGFI = 0.853; IFI = 0.957; and NFI = 0.874). The acceptable level of CFI, IFI, GFI, and AGFI is generally equal to, or greater than, 0.9. Values from 0.8 to 0.9 are considered marginal. RMSEA is acceptable between 0.03 and 0.08 and relative Chi-square (CMIN/DF) equal to, or less than, three is associated with a better fit (54). Thus, the results of our measurement model commonly show an adequate fit.

To confirm convergent validity, the average variance extracted (AVE) and composite reliability (CR) were calculated. AVE results show that the value of this index for all variables studied is more than 0.5. Except for perceived severity, perceived benefits, and behavior, the composite reliability calculated for the model variables was >0.7 (Table 2). The acceptable value for AVE and CR is 0.5 and 0.7, respectively (54). However, according to (55), if the composite reliability is >0.6, an AVE <0.5 is acceptable. Therefore, the instrument has an acceptable convergent validity.

### Assessment and Results of Structural Equation Model

**Figure 2:**
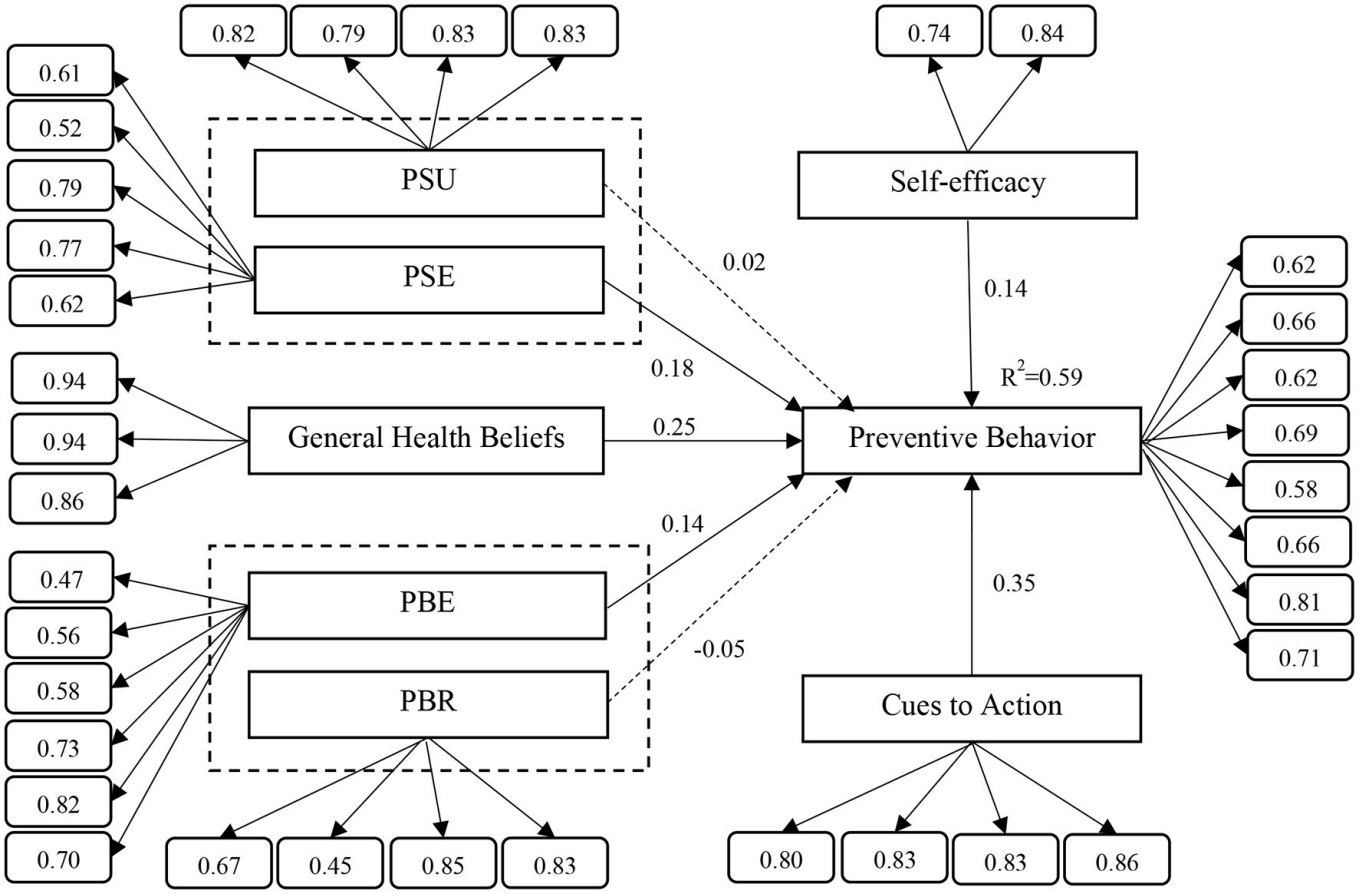
The results of the structural equation modeling (own design; factor loading of each items placed in the small boxes).

The process of validation and the structural equation model reveal sturdiness for the practical data, thus meeting the necessities of convinced indexes. The goodness-of-fit indices suggested that the HBM model has a suitable fit (RMSEA = 0.038; CMI*N* = 796.705, DF = 550, *p* = 0.000; CMIN/DF = 1.449; CFI = 0.957; GFI = 0.878; AGFI = 0.853; IFI = 0.957; and NFI = 0.874). In Table 3, it is shown that 5 out of 7 hypotheses are confirmed: Hypotheses H1 and H4 are not empirically confirmed. In the HBM model, the variables of self-efficacy (β = 0.141, *p* < 0.05), general health beliefs (β = 0.248, *p* < 0.0001), PSE (β = 0.177, *p* < 0.05), PBE (β = 0.142, *p* < 0.05), and cue to action (β = 0.347, *p* < 0.0001) have a significant impact on behavior and are able to predict 59% of behavioral changes (Figure 2).

**Table 3:** The structural model results.

**Hypothesis**	**Paths**	**Coefficient**	**Standard error**	***t*-value**	**Results**
H1	Perceived Susceptibility → Behavior	0.018	0.012	0.294	Not supported
H2	Perceived Severity → Behavior	0.177	0.169	2.156	Supported
H3	Perceived Benefits → Behavior	0.142	0.153	2.179	Supported
H4	Perceived Barriers → Behavior	0.047	0.052	0.861	Not supported
H5	Perceived Self-Efficacy → Behavior	0.141	0.044	2.287	Supported
H6	Cue to action → Behavior	0.347	0.273	4.784	Supported
H7	General Health beliefs → Behavior	0.248	0.050	4.003	Supported

*t-value > 1.96 indicates significance*.

## Discussion

Effective suppression of COVID-19 requires nontrivial efforts by a substantial portion of the world's population, including social distancing and preventing unnecessary meetings with others. No matter how strongly these actions suggested, some individuals may not trust that they can or should follow these recommendations (8). For effective policymaking to prevent the spread of the disease, it is crucial to know how beliefs affect the adoption of preventive measures. Using HBM, an approach used to exam behaviors related to disease prevention or reduction this study answers this question. Here, the relationship between health beliefs and preventive behaviors is investigated using HBM.

Results show that HBM predicts a significant percentage (59%) of preventive behavior, which is high in the psychological and behavioral literature. The results of the study also show that the cue to action, general health beliefs, PSE, PBE, and self-efficacy can predict preventive behaviors. However, PSU and PBR do not have a significant impact on preventive behavior.

According to the findings, among the variables of threat assessment, the perceived severity variable is a positive predictor of protective behaviors (Hypothesis H1). If a person feels that getting COVID-19 disease will profoundly affect their life, they are more likely to engage in protective behaviors. Akter (6) points out that some people do not take the COVID-19 pandemic seriously. Whether or not people engage in preventive activities depends on how seriously they take the consequences of the disease. The results are consistent with existing studies (13, 56–58).

Based on the findings, understanding the benefits of performing preventive behavior significantly affects that behavior (Hypothesis H3). People do not change their behavior until they receive something in return for doing so (6).

The more respondents feel that the recommended behaviors (home quarantine and the use of safeguards) effectively prevent coronavirus infection, the more likely they are to implement the behaviors. In other words, if a person believes in the benefits of preventive activities to reduce the risks, they are more willing to engage in them. This result is constant with the existing literature (44, 45, 56, 57).

Perceived self-efficacy is another predictor of protective behaviors (Hypothesis H5). Self-efficacy is an individual's belief in his or her capability to change performance (47); here, the extent to which a person feels that he or she can apply protective methods. If a person believes in their ability to access and use protective equipment, they are likely to engage in protective behavior. This result is consistent with previous studies (22, 44, 45, 57, 59). The cue to action is the most important predictor of people's protective behavior (hypothesis H6). Cue to action is a stimulus that motivates people to take action to prevent the transmission of the disease. These stimuli can be clips or news about the death of people due to COVID-19 or the effects and consequences of this disease on the lives of people, whether via mass media or social networks (6). Seeing or hearing about any of these (cues to action) can encourage people to take protective measures. These findings are consistent with previous studies (56, 57, 60). The present study provides evidence of the effectiveness of public health beliefs in performing preventive behaviors (Hypothesis H7). Public health beliefs refer to a person's values, beliefs, concerns, and readiness for health issues in daily activities. People who are aware of health issues and choose a wellness-oriented routine are more likely to engage in preventative actions than people who are unaware of health issues (61). In this study, young people who have a higher level of awareness about health measures and their impact on health are more likely to engage in preventive behaviors. The effect of PSU and PBR are not significant in the preventive behaviors of rural youth (Hypothesis H2, H4).

### Policy Implications

The present study provides important results for the development of policies and guidelines to avoid the further spread of COVID-19. Officials should inform rural youth of the severe consequences of the disease through enhanced news or the provision of educational materials (guidelines and recommendations). Young people need to know that, although the disease is incurable, they can prevent the severe consequences of the disease and reduce its damage. Policies and programs encouraging rural youth to engage in preventive behaviors should include education through mass media, social media, posters, and other advertisements.

Given the importance of self-efficacy, agricultural promotion experts, local associations, councils, and local trustees can talk to rural youth about the ease with which preventative behaviors can be practiced, thus building the confidence and beliefs of rural youth that they can effectively implement preventive measures to prevent disease. Given the vital role of perceived benefits in implementing preventive behavior, the extension efforts should highlight the benefits of preventive behavior, such as protecting not just their own lives but also their loved ones. To improve the efficacy of prevention efforts, extension experts and health officials need to strengthen public health beliefs regarding COVID-19 through information and awareness campaigns that use publicity and educational materials on preventive activities. Finally, we believe that COVID-19 will create a new role for agricultural extension services in developing countries like Iran. Most variables affecting preventive behavior are triggered by education and awareness; thus, training and awareness about the depth of risk and seriousness of the disease, public health beliefs, stimulants (cues/trigger), and the benefits of preventive behaviors along with the ability of young people to perform these actions are fundamental and necessary. As extension agents have been working in rural areas for years, educating and working with villagers, the simplest and cheapest way to achieve broad outreach in rural areas is to equip extension agents with the materials needed to teach villagers how to deal with COVID-19.

## Conclusion

The adoption of preventive measures varies across regions, socio-economic status, and age of people. Understanding behavioral differences is crucial for better targeting education, campaigns, and policies toward different groups. Only if the needs of different groups are properly accounted for will adoption rates be enhanced and the spread of diseases such as COVID-19 limited. In addition, people's perceptions are very influential in a rapid change in their behavior toward COVID-19. This study investigates the factors affecting the preventive behavior of rural youth in relation to the COVID-19 pandemic using HBM. The results using structural equations show good explanatory power for self-reported behavior. The HBM model predicts 59% of the variance of preventive behavior against COVID-19 outbreaks, such as wearing gloves and masks, regular hand washing, and regular hand disinfection. All of HBM's independent constructs, except perceived susceptibility and perceived barriers affect preventive behavior. The results show that cue to action is the strongest predictor of behavior.

This study adds new and important knowledge to existing information on the effectiveness of HBM in predicting the preventive behaviors of rural youth against COVID-19. The results of this study are useful for designing better policies and public information campaigns on measures to contain the spread of health diseases such as COVID-19. Further, the results of this study can be used as a basis for further studies on the COVID-19 pandemic. This theory could also be a roadmap for modifying adaptive behaviors to help effectively manage unknown pandemics in the future.

## Limitations and Directions for Future Research

While this study is valuable information to the body of research on COVID-19, it is not without its limitations. This study is a cross-sectional study and its data was collected in August 2020 in Iran, so caution should be exercised in generalizing the results to other regions and times. Another potential limitation of this study was that the questionnaire was online and as a result, the respondents were the only people who had access to the Internet. While not all villagers have high access to the Internet. Therefore, it is possible that some villagers are less likely to participate in data collection. Another limitation is that behaviors are self-reported. The self-report does not allow the assessment of actual behavior (35). It is suggested that in future studies, the actual behaviors of individuals be examined. In addition, the paradigm of this study is quantitative. It is suggested that combined quantitative-qualitative methods be used in future studies to obtain more accurate results.

## Data Availability Statement

The raw data supporting the conclusions of this article will be made available by the authors, without undue reservation.

## Ethics Statement

Ethical review and approval was not required for the study on human participants in accordance with the local legislation and institutional requirements. The patients/participants provided their written informed consent to participate in this study.

## Author Contributions

MT, TZ, KL, and SS contributed to conception and design of the study. MT and TZ conducted the data collection, performed the statistical analysis, and wrote the first draft of the manuscript. KL and SS wrote sections of the manuscript. All authors contributed to manuscript revision, read, and approved the submitted version.

## Conflict of Interest

The authors declare that the research was conducted in the absence of any commercial or financial relationships that could be construed as a potential conflict of interest.

## Publisher's Note

All claims expressed in this article are solely those of the authors and do not necessarily represent those of their affiliated organizations, or those of the publisher, the editors and the reviewers. Any product that may be evaluated in this article, or claim that may be made by its manufacturer, is not guaranteed or endorsed by the publisher.
